# Supplementary material for: Netcombin: An algorithm for constructing optimal phylogenetic network from rooted triplets
Source: PLoS One. 2020 Sep 18;15(9):e0227842. doi: 10.1371/journal.pone.0227842 (PMC7500971; doi:10.1371/journal.pone.0227842)
Supplement: S4 File — (ZIP) [file pone.0227842.s004.zip › SequenceData-Triplet/sequenceData-10-20-30-0-Triplet/length 1000, numseq 40, rate 10^-7/1 seq.pdf]

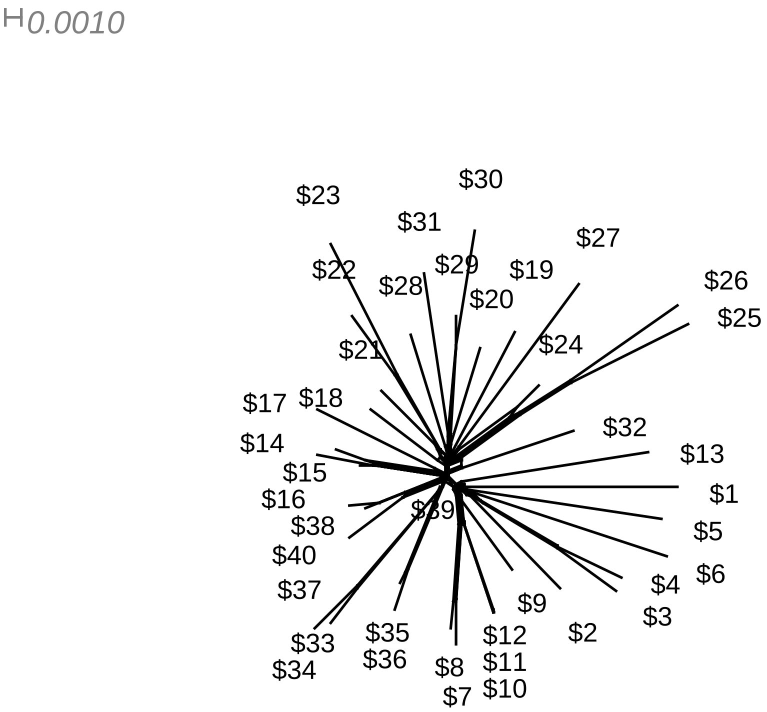
0.0010
$30
$23
$31
$27
$29
$22
$19
$28
$20
$26
$25
$21
$24
$17
$18
$32
$14
$13
$15
$1
$16
$39
$5
$38
$40
$6
$4
$37
$9
$3
$2
$35
$12
$33
$36
$11
$34
$8
$10
$7
